# Angiosarcoma Arising from the Main Pulmonary Artery Mimicking Pulmonary Thromboembolism

**DOI:** 10.5935/abc.20170077

**Published:** 2017-07

**Authors:** Joana Sofia Silva Moura Ferreira, Nádia Moreira, Maria João Ferreira, Manuel Antunes

**Affiliations:** Centro Hospitalar e Universitário de Coimbra, Portugal

**Keywords:** Echocardiography, Pulmonary Embolism, Thoracic Surgery

A 79-year-old female with no relevant past medical history was admitted in our emergency department for dyspnea on minimal exertion and chest discomfort over 2 weeks. Blood gas analysis showed severe hypoxemia and hypocapnia. Troponin was slightly positive. Despite a negative D-dimer assay, contrast-enhanced chest CT was performed to exclude pulmonary embolism. It showed a large filling defect centered in the pulmonary valve plane (Panels A and B). Bedside transthoracic echocardiogram showed a large echodense mass, apparently mobile, extending across the right ventricle outflow tract, pulmonary valve, and the main pulmonary artery, with dilatation of the right sided chambers and transtricuspid peak gradient of 70 mmHg (Panels C and D). Lower-limb venous compression ultrasound was negative for deep vein thrombosis. The patient remained stable, but required high oxygen inspiration fraction to maintain saturation above 90%. As pulmonary embolism was deemed unlikely given the clinical findings, the patient underwent cardiac surgery. Surgery revealed a pearly mass in the main pulmonary artery obliterating almost the entire lumen and with upstream extension to the pulmonary valve and right ventricle outflow tract (Panel E). The tumor was excised as much as possible and the pulmonary valve was replaced by a homograft. Pathological examination was compatible with angiosarcoma.

Pulmonary artery angiosarcoma is exceedingly rare and carries a very poor prognosis. It can be clinical and radiologically indistinguishable from acute or chronic pulmonary artery thromboembolism. Our clinical suspicion was heightened by a negative D-dimer assay and venous ultrasound and the apparent infiltration of pulmonary arterial walls on CT.

**Figure 1:**
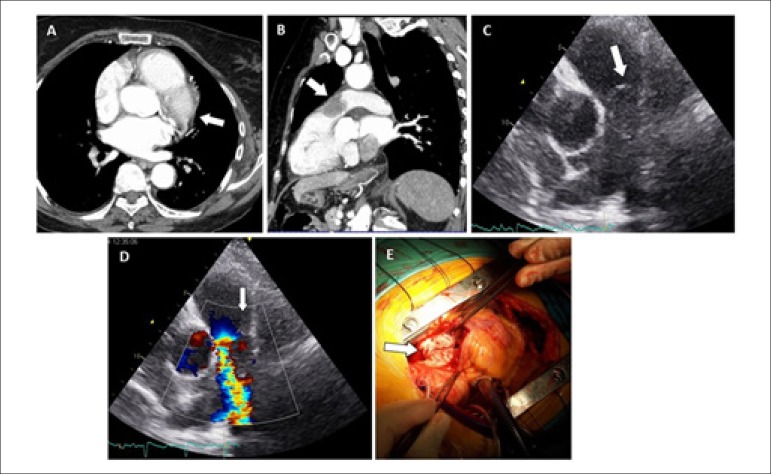
Angiosarcoma.

